# Behavioral and steroidogenic pharmacology of phenyl ring substituted etomidate analogs in rats

**DOI:** 10.1186/s40360-019-0328-4

**Published:** 2019-08-05

**Authors:** Megan McGrath, Alissa Hofmann, Douglas E. Raines

**Affiliations:** Department of Anesthesia Critical Care and Pain Medicine, Massachusetts General Hospital, 55 Fruit Street, GRB444, Boston, MA 02114 USA

**Keywords:** Anesthetic, Corticosterone, Cushing’s syndrome, Etomidate, Hypercortisolemia, Steroidogenesis

## Abstract

**Background:**

Cushing’s syndrome is an endocrine disorder characterized by the overproduction of adrenocortical steroids. Steroidogenesis enzyme inhibitors are the mainstays of pharmacological treatment. Unfortunately, they produce significant side effects. Among the most potent inhibitors is the general anesthetic etomidate whose GABA_A_ receptor-mediated sedative-hypnotic actions restrict use. In this study, we defined the sedative-hypnotic and steroidogenesis inhibiting actions of etomidate and four phenyl-ring substituted etomidate analogs (dimethoxy-etomidate, isopropoxy-etomidate, naphthalene-etomidate, and naphthalene (2)-etomidate) that possess negligible GABA_A_ receptor modulatory activities.

**Methods:**

In the first set of experiments, male Sprague-Dawley rats were assessed for loss of righting reflexes (LoRR) after receiving intravenous boluses of either etomidate (1 mg/kg) or an etomidate analog (40 mg/kg). In the second set of experiments, rats were assessed for LoRR and their abilities to produce adrenocortical and androgenic steroids after receiving 2-h infusions (0.5 mg kg^− 1^ min^− 1^) of either etomidate or an etomidate analog.

**Results:**

All rats that received etomidate boluses or infusions had LoRR that persisted for minutes or hours, respectively. In contrast, no rat that received an etomidate analog had LoRR. Compared to rats in the vehicle control group, rats that received etomidate analog infusions had plasma corticosterone and aldosterone concentrations that were reduced by 80–84% and 68–94%, respectively. Rats that received etomidate infusions had plasma corticosterone and aldosterone concentrations that were also significantly reduced (by 92 and 96%, respectively). Rats that received etomidate or isopropoxy-etomidate had significant reductions (90 and 57%, respectively) in plasma testosterone concentrations whereas those that received naphthalene-etomidate had significant increases (1400%) in plasma dehydroepiandrosterone concentrations. Neither etomidate nor any etomidate analog significantly affected plasma androstenedione and dihydrotestosterone concentrations.

**Conclusions:**

Our studies demonstrate that the four phenyl-ring substituted etomidate analogs form a novel class of compounds that are devoid of sedative-hypnotic activities and suppress plasma concentrations of adrenocortical steroids but vary in their effects on plasma concentrations of androgenic steroids.

## Background

Cushing’s syndrome is a rare endocrine disorder that is characterized by the overproduction of adrenocortical steroids and associated with significant morbidity including insulin resistance, hypertension, immunosuppression, cardiovascular disease, neurocognitive deficits, and early mortality [1–6]. It is most commonly due to the presence of an adrenocorticotropic hormone (ACTH)-secreting pituitary tumor, but may also be caused by an adrenocortical adenoma, carcinoma, or hyperplasia. Surgical intervention is typically the treatment of choice; however, recurrence after surgery is common, necessitating long-term pharmacological management to suppress cortisol production [7–12]. Pharmacological management may also be administered preoperatively to reduce surgical risk, to treat metastatic disease, or combined with radiotherapy as an alternative or in addition to surgery [13, 14].

**Fig. 1:**
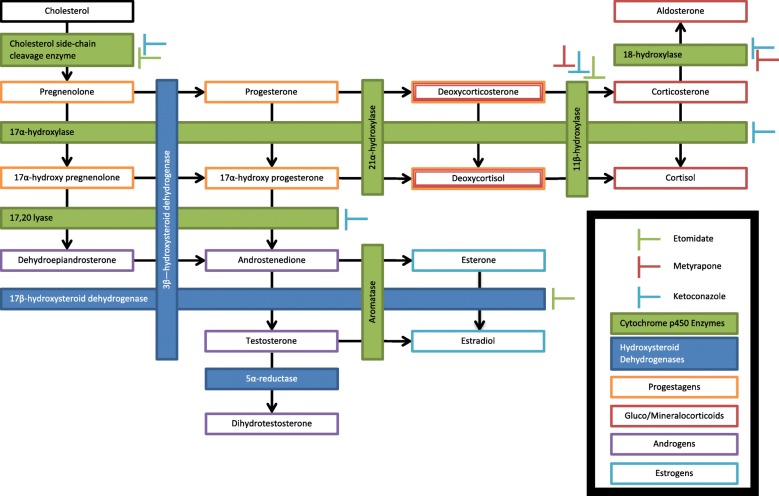
A graphic representation of the steroidogenic pathways. As indicated in the figure inset, filled boxes represent enzymes, with green and blue boxes indicating cytochrome P450 and hydroxysteroid dehydrogenase superfamily enzymes, respectively. Orange boxes indicate progestogens whereas dark red boxes indicate the gluco- and mineral-corticoids. Purple and light blue boxes indicate androgens and estrogens, respectively. The proposed inhibitory sites of common steroidogenesis inhibitors are shown with green, red, and light blue bars, representing etomidate, metyrapone, and ketoconazole, respectively

Although several classes of drugs can be utilized in the treatment of Cushing’s syndrome, steroidogenesis enzyme inhibitors are the mainstays of pharmacological management [15]. Steroidogenesis inhibitors suppress cortisol production by inhibiting enzymes required for the biosynthesis of adrenocortical steroids (Fig. 1). These enzymes fall into one of two structurally related superfamilies, cytochrome P450s and hydroxysteroid dehydrogenases. The most commonly employed steroidogenesis enzyme inhibitors are ketoconazole and metyrapone, although aminoglutethimide, mitotane, and fluconazole are also sometimes used [15]. Unfortunately, all of these drugs have significant adverse side effects that limit dosing, clinical effectiveness, and patient tolerability, and none have been approved by the United States Food and Drug Administration for the treatment of Cushing’s syndrome. Consequently, there are ongoing efforts to develop new drugs with improved pharmacological profiles [16].

**Fig. 2:**
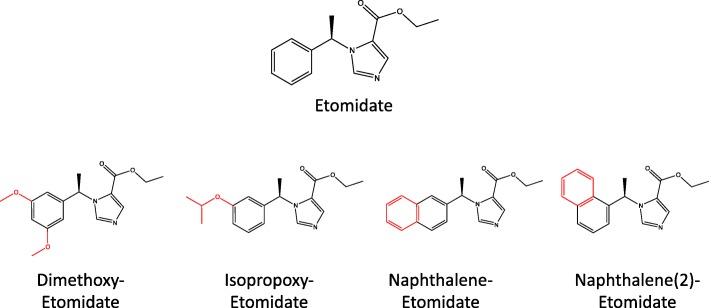
Chemical structures of etomidate and the four etomidate analogs examined. Benzene ring modifications for each analog are highlighted in red

Etomidate is a general anesthetic induction agent that exerts its sedative-hypnotic actions via its positive modulatory actions on the GABA_A_ receptor [17]. It is also used “off-label” to treat Cushing’s syndrome because it potently inhibits the function of the adrenocortical CYP enzyme 11β-hydroxylase and potently suppresses the synthesis of cortisol [18–20]. Within the context of its use as a steroidogenesis enzyme inhibitor, the GABA_A_ receptor modulatory activity and resultant sedative-hypnotic actions of etomidate are undesirable side effects that have led to recommendations that etomidate administration be restricted to highly monitored hospital environments such as an intensive care unit [15, 20–22]. These side effects are most likely to be encountered when employing a “block and replace” strategy where the goal is to completely inhibit cortisol biosynthesis (and then to administer replacement corticosteroids) because relatively high etomidate doses are required. As part of a strategy to overcome this limitation we developed (*R*)-ethyl 1-(1-(3,5-dimethoxyphenyl)ethyl)-1*H*-imidazole-5-carboxylate (dimethoxy-etomidate), a phenyl ring substituted etomidate analog that retains the potent 11β-hydroxylase inhibitory activity of etomidate but is essentially devoid of the drug’s GABA_A_ receptor modulatory and sedative-hypnotic actions [23]. In the current manuscript, we describe studies to further define the pharmacology of this compound along with three additional phenyl ring substituted etomidate analogs that are similarly lacking in significant GABA_A_ receptor modulatory activity (Fig. 2) [24]. We also characterized the pharmacology of etomidate to provide context. Our studies show that these phenyl ring substituted etomidate analogs form a novel class of compounds that retain the ability of etomidate to suppress adrenocortical steroid biosynthesis but do not have sedative-hypnotic activity. However, despite their similar inhibitory actions on adrenocortical steroid biosynthesis, these etomidate analogs variably affect the biosynthesis of individual androgenic steroids.

## Methods

### Sources of drugs and chemicals

ACTH_,_ human chorionic gonadotropin hormone (hCG), and Glycine-HCl were purchased from Sigma-Aldrich Chemical Company (St. Louis, MO, USA). Isoflurane was purchased from Patterson Veterinary (Saint Paul, MN, USA). Heparin and saline were purchased from Hospira (Lake Forest, IL, USA). EDTA-containing vacutainer tubes were purchased from Becton Dickinson (Franklin Lakes, NJ, USA). Etomidate was purchased from Bachem (Torrance, CA, USA). Enzyme-linked immunosorbent assay kits for corticosterone were purchased from IDS (Gaithersburg, MD, USA), whereas those for aldosterone, dehydroepiandrosterone (DHEA), and testosterone were purchased from Enzo Life Sciences (Farmingdale, NY, USA), and those for androstenedione and dihydrotestosterone were purchased from MyBioSource (Cambridge, MA, USA). Dimethoxy-etomidate, (*R*)-ethyl 1-(1-(3-isopropoxyphenyl)ethyl)-1*H*-imidazole-5-carboxylate (isopropoxy-etomidate), (*R*)-ethyl 1-(1-(naphthalen-2-yl)ethyl)-1*H*-imidazole-5-carboxylate (naphthalene-etomidate), and (*R*)-ethyl 1-(1-(naphthalen-1-yl)ethyl)-1*H*-imidazole-5-carboxylate (naphthalene (2)-etomidate) were custom synthesized by Aberjona Laboratories (Beverly, MA, USA) and their structures confirmed by nuclear magnetic resonance spectroscopy.

### Animals

All studies were conducted with the approval of and in accordance with rules and regulations of the Institutional Animal Care and Use Committee at the Massachusetts General Hospital, Boston, Massachusetts (Protocol # 2013 N000130) and the principles outlined in the Guide for the Care and Use of Laboratory Animals from the National Institutes of Health. Adult male Sprague-Dawley rats (300–500 g) were purchased from Charles River Laboratories (Wilmington, MA, USA) and housed with a 12–12 light-dark cycle with light switched on at 7 AM. Rats were caged in our animal care facility, which is maintained at 22.5 °C, and allowed free access to food and drinking water until the day of study. Rats were housed socially (i.e. with other rats) whenever possible with access to enrichment materials (wooden block and plastic tube). Each rat was weighed on the day of study. All studies were performed in our laboratory at the Massachusetts General Hospital and carried out at room temperature. During infusions, animal core temperature was monitored and maintained at a target temperature of 36–37 °C using a heat lamp. At the end of study, all animals were euthanized by our veterinary staff in the animal care facility by carbon dioxide asphyxiation or in our laboratory under propofol anesthesia using intravenous KCl as recommended under American Veterinary Medical Association guidelines.

### Etomidate and etomidate analog formulation, dosing and administration

For experiment 1, our goal was to test all etomidate analogs for sedative-hypnotic activity at the highest attainable intravenous bolus dose that was reliably non-lethal. That dose was determined in pilot studies to be 40 mg kg^− 1^. An etomidate intravenous bolus dose of 1 mg kg^− 1^ was chosen for context with the expectation that it would produce loss of righting reflexes in all rats [25]. On the day of study, rats were blocked randomized into 6 rats per treatment group (i.e. etomidate group, dimethoxy-etomidate group, naphthalene-etomidate group, naphthalene (2)-etomidate group). To allow intravenous dosing, a 24-gauage catheter was placed in a tail vein under brief isoflurane (2–3%) anesthesia. This method of anesthesia was chosen to allow rapid reversibility. Catheter patency was maintained with a heparin lock flush. Animals were then allowed to recover for at least 2 hours prior to study. Etomidate and etomidate analogs were formulated for these bolus studies using Captisol (Ligand, San Diego, CA) at a 1:1 M ratio in distilled water at concentrations of 2 mg/ml and 50 mg/ml, respectively.

For experiment 2, our primary goal was to assess the impact of 2-h etomidate and etomidate analog infusions on steroidogenesis. Our secondary goal was to determine whether such infusions produce sedation/hypnosis. For these studies, we used the highest attainable dose (0.5 mg kg^− 1^ min^− 1^) given the limitations in etomidate and analog aqueous solubility and infusion volume constraints in rats. On the day of study, rats were block randomized into 6 rats per treatment group (i.e. vehicle control group, etomidate group, dimethoxy-etomidate group, naphthalene-etomidate group, naphthalene (2)-etomidate group). Two 24-gauge intravenous catheters were placed in each rat (one in each lateral tail vein) under brief isoflurane (2–3%) anesthesia to allow the reliable intravenous administration of drugs and test compounds and to draw blood. Catheter patency was maintained with a heparin lock flush. Animals were then allowed to recover for at least 2 hours prior to study. To avoid potential interference with our steroid assays by Captisol [26, 27], etomidate and etomidate analogs were formulated for these infusion studies on the study day in 10 mM glycine-HCl buffer (pH 2–2.2) at a concentration of 3 mg/ml.

### Loss of righting reflex by etomidate and etomidate analogs

Immediately after receiving an etomidate or etomidate analog bolus (Experiment 1) or at the end of a 2-h etomidate or etomidate analog infusion (Experiment 2), each rat was assessed for sedation/hypnosis using a loss of righting reflex assay [28]. In this assay, a rat is determined to have lost its righting reflexes if (after being turned supine) it fails to turn itself back onto all four paws within 5 s. Each rat that has loss of righting reflexes is then monitored and the duration of loss of righting reflexes defined as the time when etomidate or etomidate analog administration was complete until the animal returned onto all four paws.

### Steroidogenesis inhibition by etomidate and etomidate analogs

**Fig. 3:**
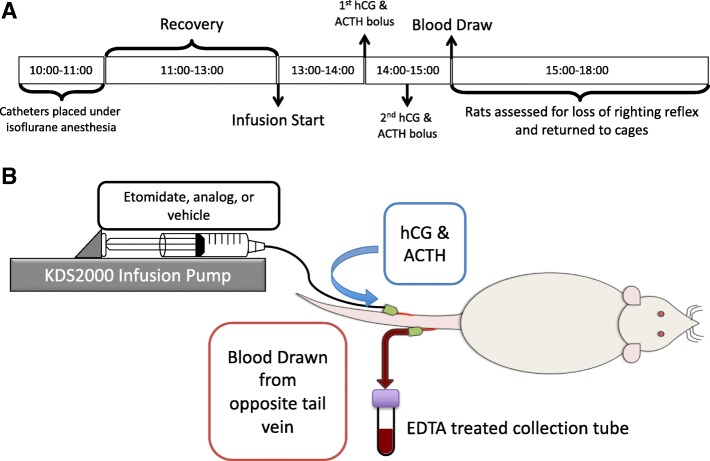
Graphic representation of the experimental protocol. **a** Boxes show the protocol on experimental days. **b** A graphic representation of the experimental set-up

In Experiment 2, the in vivo steroidogenic actions of etomidate and etomidate analogs were assessed in male rats (six per group) using a hormone stimulation test (Fig. 3). For the 2 days immediately prior to study, rats were restrained in a broome style rodent restrainer from 10 AM until 4 PM to accommodate them to this environment and reduce stress-induced fluctuations in steroid biosynthesis. On the study day, each rat was placed in a restrainer at 10 AM and intravenous catheters were placed as described above. At 1 PM, a 2-h infusion of 0.5 mg/kg/min etomidate, etomidate analog, or an equivalent volume of 10 mM Glycine-HCl, pH 2.2 vehicle was started. At the timepoints indicated in Fig. 3, adrenocortical and androgenic steroid biosynthesis was stimulated in rats by administering intravenous ACTH (25 μg/kg) and hCG (10 U/kg), respectively. At the end of the infusion, a blood sample was drawn and rats were assessed for loss of righting reflexes as described in the following section. Stimulated plasma steroid concentrations were determined using enzyme-linked immunosorbent assays and a 96-well plate reader (Molecular Devices, Sunnyvale, CA).

### Statistical analysis

All individual data points are expressed as the mean ± the S.E.M. of six independent measurements. Group size was chosen based on previous experience [23]. Statistical analyses to assess differences in plasma steroid concentrations between etomidate or etomidate analog groups versus the vehicle control groups were carried out with a Mann-Whitney U test using Prism 5 for Mac OS X. A Bonferroni correction was used to account for multiple (i.e. five) comparisons. Thus, statistical significance was assumed for a *p* < 0.01.

## Results

### Experiment 1

#### Sedative-hypnotic actions of etomidate and etomidate analogs following bolus administration

All rats that received an etomidate bolus (1 mg/kg; *n* = 6 rats) exhibited loss of righting reflexes. This loss of righting reflexes lasted for 3.4 ± 0.3 min. In contrast, none of the rats that received an etomidate analog bolus (40 mg/kg; n = 6 rats × 4 analogs) exhibited loss of righting reflexes.

### Experiment 2

#### Sedative-hypnotic actions of etomidate and etomidate analogs following 2-h infusion

All 6 rats that received etomidate exhibited loss of righting reflexes when assessed at the end of the 2-h etomidate infusion. Such loss of righting reflexes persisted for 152 ± 3 min (range: 146–157 min). In contrast, none of the 24 rats that received a 2-h infusion of any of the four etomidate analogs (or the 6 that received vehicle alone) exhibited loss of righting reflexes.

#### Impact of etomidate and etomidate analogs on plasma adrenocortical steroid concentrations

**Fig. 4:**
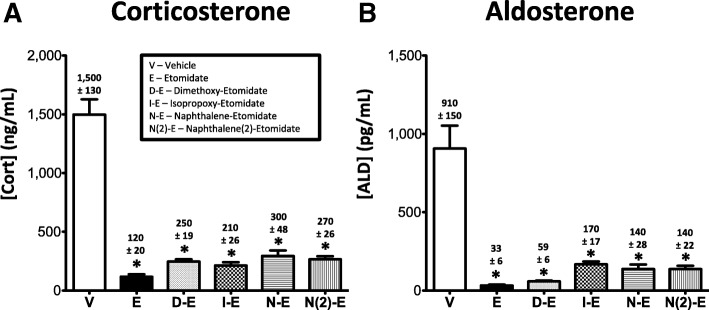
Adrenocorticotropic hormone-stimulated plasma concentrations of (**a**) corticosterone and (**b**) aldosterone following administration of vehicle or 0.5 mg/kg/min IV etomidate, dimethoxy-etomidate, isopropoxy-etomidate, naphthalene-etomidate, or naphthalene (2)-etomidate to rats. Abbreviations for each test compound are defined in panel A. Each bar represents the mean ± SEM from 6 rat experiments. * *p* < 0.01

Consistent with our previous studies [23, 29, 30], both etomidate and dimethoxy-etomidate significantly reduced ACTH-stimulated plasma corticosterone concentrations in rats by 92 and 84%, respectively, as compared to those concentrations measured in vehicle control group rats. We similarly found that the three new phenyl ring substituted etomidate analogs isopropoxy-etomidate, naphthalene-etomidate, and naphthalene (2)-etomidate significantly reduced such concentrations by 86, 80, and 82%, respectively (Fig. 4a). Etomidate and all four of the etomidate analogs also significantly reduced ACTH-stimulated plasma aldosterone concentrations in rats by 96% (etomidate), 94% (dimethoxy-etomidate), 76% (isopropoxy-etomidate), 68% (naphthalene-etomidate) and 71% (naphthalene (2)-etomidate) as compared to those concentrations measured in vehicle control group rats (Fig. 4b).

#### Impact of etomidate and etomidate analogs on plasma androgenic steroid concentrations

**Fig. 5:**
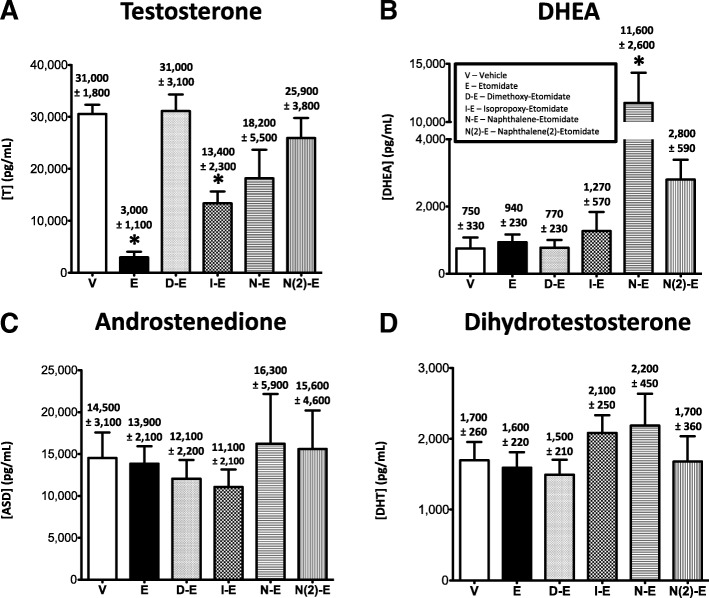
Human Chorionic Gonadotropin-stimulated plasma concentrations of (**a**) testosterone, (**b**) dehydroepiandrosterone (DHEA), (**c**) androstenedione, and (**d**) dihydrotestosterone following administration of vehicle or 0.5 mg/kg/min IV etomidate, dimethoxy-etomidate, isopropoxy-etomidate, naphthalene-etomidate, or naphthalene (2)-etomidate to rats. Abbreviations for each test compound are defined in panel B. Each bar represents the mean ± SEM from 6 rat experiments. * p < 0.01

In contrast to their similar inhibitory actions on stimulated plasma concentrations of the two adrenocortical steroids (corticosterone and aldosterone), etomidate and the four etomidate analogs varied in their impact on stimulated plasma concentrations of the androgenic steroids testosterone (Fig. 5a) and dehydroepiandrosterone (Fig. 5b) and had no significant effect on plasma concentrations of androstenedione (Fig. 5c) and dihydrotestosterone (Fig. 5d). Specifically, etomidate significantly reduced hCG-stimulated plasma testosterone concentrations in rats by an order of magnitude from a vehicle control value of 31,000 ± 1800 pg/mL to 3000 ± 1100 pg/mL whereas dimethoxy-etomidate had no effect at all (31,000 ± 3100 pg/mL). The other three etomidate analogs modestly reduced plasma testosterone concentrations in hCG-stimulated rats to values of 13,400 ± 2300 (isopropoxy-etomidate), 18,200 ± 5500 (naphthalene-etomidate), and 25,900 ± 3800 (naphthalene (2)-etomidate). However, only the reduction by isopropoxy-etomidate reached statistical significance (Fig. 5a).

Both naphthalene-etomidate and naphthalene (2)-etomidate increased hCG-stimulated plasma DHEA concentrations in rats from a vehicle control value of 750 ± 330 pg/mL to values of 11,600 ± 2600 pg/mL and 2800 ± 590 pg/mL, respectively. In the case of naphthalene-etomidate, this increase (1400%) reached statistical significance. hCG-stimulated plasma DHEA concentrations in rats that received etomidate (940 ± 230 ng/mL), dimethoxy-etomidate (770 ± 230 ng/mL), and isopropoxy-etomidate (1270 ± 570 ng/mL) were not significantly different from those in the vehicle control group (Fig. 5b).

The hCG-stimulated plasma concentrations of androstenedione and dihydrotestosterone in control rats that had received vehicle alone were 14,500 ± 3100 pg/mL and 1700 ± 260 pg/mL, respectively. Neither etomidate nor any of the etomidate analogs studied had any significant effect on hCG-stimulated plasma concentrations of these two androgens (Figs. 5c & d).

## Discussion

In the current manuscript, we report the results of animal studies aimed at further defining the pharmacology of dimethoxy-etomidate – a potential lead compound for the development of new drugs to treat Cushing’s syndrome – and three new etomidate analogs that also contain bulky phenyl-ring substituent groups and are essentially devoid of GABA_A_ receptor modulatory activity. In addition, we describe the results of studies using the sedative-hypnotic etomidate to provide context. We show that the four phenyl-ring substituted etomidate analogs form a novel class of etomidate analogs that (1) unlike etomidate, are essentially devoid of sedative-hypnotic activities; (2) similar to etomidate, suppress plasma concentrations of adrenocortical steroids; and (3) vary in their effects on plasma concentrations of androgenic steroids.

Steroid biosynthesis is primarily mediated by enzymes belonging to the cytochrome p450 and hydroxysteroid dehydrogenase superfamilies. The structures of the enzymes that comprise each of these superfamilies are highly homologous, explaining the low selectivities that most exogenous ligands (e.g. steroidogenesis inhibitors) possess for superfamily members [31]. For example, ketoconazole inhibits 17α-hydroxylase, cholesterol side chain cleavage enzyme, 11β-hydroxylase, 18-hydroxylase, and 17,20-lyase [15]. At clinical doses, metyropone inhibits both 11β-hydroxylase and the closely related enzyme 18-hydroxylase [15]. For each steroidogenesis inhibitor, the specific pattern of enzyme inhibition determines whether it will cause the plasma concentration of a particular steroid to rise or fall. Because ketoconazole inhibits enzymatic steps that are relatively proximal in the steroidogenesis pathway and thus required for the biosynthesis of both adrenocortical and androgenic steroids, it reduces the plasma concentrations of steroids from both classes (e.g. cortisol and testosterone, respectively). This leads to side effects in male patients that are attributable to low androgen levels such as decreased libido and gynecomastia [13, 32, 33]. In contrast, the two enzymes inhibited by metyropone are near the end of the steroidogenesis pathway and required only for adrenocortical steroid biosynthesis. Inhibition of these two enzymes selectively reduces adrenocortical steroid production and causes adrenocortical steroid precursors to accumulate. These precursors may then be shunted into the androgen (and mineralcorticoid) steroid biosynthetic pathway, increasing androgenic steroid levels and causing such side effects as acne, hirsutism, virilism, hypertension, and infertility [13, 34–37].

As expected, we found that all rats that received a 1 mg/kg etomidate bolus or 2-h etomidate infusion exhibited loss of righting reflexes, an animal correlate for sedation/hypnosis. In contrast, none of the rats that received a 40-fold higher bolus dose of an etomidate analog (i.e. 40 mg/kg) or a 2-h etomidate analog infusion lost their righting reflexes. Such a lack of sedative-hypnotic activity among the etomidate analogs is consistent with previous electrophysiological work showing that these analogs are essentially devoid of the GABA_A_ receptor positive modulatory actions thought to mediate the sedative-hypnotic effects of etomidate [24, 38, 39].

Consistent with previous studies, we also found that etomidate reduces plasma concentrations of corticosterone and aldosterone [23, 29, 30]. This action has been attributed to inhibition of 11β-hydroxylase and 18-hydroxylase, mechanisms that it shares with metyrapone [15]. Molecular docking studies utilizing these two highly homologous enzymes indicate that high affinity binding of etomidate and metyrapone is mediated by a coordination bond that forms between the aromatic nitrogen found in the imidazole ring of these drugs and the heme iron located at the active sites of the two enzymes [31]. In the case of etomidate, the critical nature of this interaction has been confirmed by studies using a pyrrole etomidate analog (carboetomidate) that is unable to form such a bond; carboetomidate possesses an in vitro enzymatic binding affinity and an in vivo adrenocortical inhibitory potency that are three orders of magnitude lower than those of etomidate [40, 41]. Other important interactions identified by docking studies include a hydrogen bonding interaction between the etomidate ester moiety and Thr318 in the active site and a ring stacking interaction between the etomidate phenyl ring and Phe130 in the active site [31]. These are interactions that are expected to be maintained with all four phenyl ring substituted etomidate analogs. However, the active site cavity volumes of these two enzymes are estimated to be 360 Å^3^ (11β-hydroxylase) and 334 Å^3^ (18-hydroxylase), which are only modestly larger than the molecular volume of etomidate (269.7 Å^3^) [31]. The addition of the various bulky substituents groups to etomidate (to form our four etomidate analogs) increases molecular volume by between 39.9 Å^3^ and 52.7 Å^3^, potentially introducing steric hindrance and reducing the binding affinity to these enzymes [24]. Such a reduction in affinity could explain – at least in part – why the four phenyl ring substituent-containing analogs tended to produce smaller reductions in plasma corticosterone and aldosterone concentrations than etomidate.

Although etomidate and all four etomidate analogs significantly reduced plasma concentrations of both adrenocortical steroids, only two compounds (etomidate and isopropoxy-etomidate) significantly reduced those of any androgenic steroid. This reduction was limited to testosterone, whose plasma concentrations were reduced by 90% (etomidate) and 57% (isopropoxy-etomidate). In contrast, naphthalene-etomidate actually increased plasma concentrations of the androgenic steroid DHEA by more than an order of magnitude without significantly affecting those of the other androgenic steroids. Unfortunately, the mechanisms responsible for these actions cannot be unambiguously determined from our data. However, previous in vitro studies indicate that etomidate can inhibit 17β-hydroxysteroid dehydrogenase leading to suppression of testosterone synthesis.

## Conclusions

In conclusion, we have defined a novel class of steroidogenic enzyme inhibitors consisting of etomidate analogs that contain bulky phenyl ring substituent groups. Compounds in this class are devoid of sedative-hypnotic activity and inhibit stimulated plasma adrenocortical steroid concentrations, but differ in their effects on stimulated plasma androgenic steroid concentrations. They provide a proof-of-concept for the development of non-sedating etomidate analogs to treat Cushing’s syndrome as well as other pathologies whose clinical courses may be improved by altering steroid biosynthesis.

## Data Availability

The datasets used and/or analyzed during the current study are available from the corresponding author on reasonable request.

## Abbreviations

ACTH: Adrenocorticotropic hormone;
DHEA: Dehydroepiandrosterone
Dimethyoxy-etomidate: (*R*)-ethyl 1-(1-(3,5-dimethoxyphenyl)ethyl)-1*H*-imidazole-5-carboxylate
GABA_A_ receptor: Gamma aminobutyric acid type A receptor
hCG: Human chorionic gonadotropin hormone
Isopropoxy-etomidate: (*R*)-ethyl 1-(1-(3-isopropoxyphenyl)ethyl)-1*H*-imidazole-5-carboxylate
Naphthalene (2)-etomidate: (*R*)-ethyl 1-(1-(naphthalen-1-yl)ethyl)-1*H*-imidazole-5-carboxylate
Naphthalene-etomidate: (*R*)-ethyl 1-(1-(naphthalen-2-yl)ethyl)-1*H*-imidazole-5-carboxylate
